# MRI-based volumetric and histogram analysis of parotid and submandibular salivary glands in type 2 diabetes mellitus: a retrospective study

**DOI:** 10.1186/s12903-026-09876-1

**Published:** 2026-09-10

**Authors:** Ferit Doğan, Sedef Kotanlı, Nurbanu Sahin, Rıza Altunbaş, Hüseyin Karaaslan

**Affiliations:** 1 Department of Radiology, Faculty of Medicine, Harran University, Sanliurfa, Turkey; 2 Department of Dentomaxillofacial Radiology, Faculty of Dentistry, Harran University, Sanliurfa, 633200 Turkey; 3 Department of Internal medicine, Sanliurfa Mehmet Akif Inan Training and Research Hospital, Sanliurfa, Turkey; 4 Department of Endocrinology, Faculty of Medicine, Harran University, Sanliurfa, Turkey

**Keywords:** Type 2 diabetes mellitus, Parotid gland, Submandibular gland, Magnetic resonance imaging, Volumetric analysis, Histogram analysis

## Abstract

**Objective:**

This retrospective study aimed to evaluate the MRI-based volumetric and histogram characteristics of the parotid and submandibular salivary glands in patients with type 2 diabetes mellitus (T2DM) compared with control participants, and to investigate potential correlations between gland volumes, HbA1c levels, and disease duration.

**Materials and methods:**

Following approval from the Harran University Clinical Research Ethics Committee, magnetic resonance (MR) images of 30 patients with T2DM and 30 control participants were retrospectively analyzed. Salivary gland volumes were measured, and demographic data, HbA1c values, and diabetes duration were recorded for the T2DM group. In addition, histogram analysis was performed on T1-weighted MR images using standardized regions of interest to calculate mean grayscale values, skewness, and kurtosis parameters. Statistical analyses were conducted to compare gland volumes between groups and to assess correlations with clinical parameters.

**Results:**

There were no statistically significant differences in the volumes of the parotid and submandibular glands between the T2DM and control groups (*p* > 0.05). Additionally, no significant correlations were found between gland volumes and HbA1c levels or disease duration within the diabetic group. Patients with T2DM demonstrated significantly lower mean grayscale values across all major salivary glands compared with controls (all *p* < 0.01), whereas skewness and kurtosis values remained comparable between groups (all *p* > 0.05).

**Conclusion:**

The findings suggest that T2DM was not associated with significant volumetric differences in the major salivary glands in this study population. However, significantly lower mean T1-weighted grayscale values were observed in patients with T2DM, whereas skewness and kurtosis remained comparable between groups. These signal-intensity differences should be regarded as exploratory imaging findings rather than direct evidence of specific microstructural alterations, and their functional and clinical significance remains uncertain. Further studies incorporating quantitative MRI techniques and functional correlation are needed to clarify their biological and clinical relevance.

## Introduction

Diabetes mellitus (DM) is a chronic metabolic disease resulting from insulin deficiency or insulin resistance and can affect multiple systems throughout the body [1]. As of 2025, approximately 11.1% of the adult population, equating to one in nine adults, is living with DM. More than 40% of these individuals are unaware of their condition, and the majority of cases (81%) are found in low- and middle-income countries. Based on current projections, it is estimated that the number of adults with DM will reach 853 million by 2050 [2, 3]. The progressively increasing global prevalence of DM necessitates a detailed investigation of its systemic effects. The vascular, neuropathic, and immunological changes caused by DM in the long term have significant impacts on many organs and tissues, primarily the cardiovascular and renal systems [2, 4]. One of these systemic effects involves the salivary glands, as DM has been shown to lead to structural and functional alterations in the parotid and submandibular glands [5, 6].

The parotid and submandibular glands are responsible for a major portion of saliva production and play a critical role in maintaining oral homeostasis [7]. Previous studies have reported decreased salivary flow rate, alterations in salivary composition (increased glucose and electrolytes, decreased protein and immunological components), and oral complications such as dry mouth (xerostomia), dental caries, and periodontal diseases in patients with DM [1, 5, 6, 8]. Studies have suggested that morphological and volumetric changes may occur in the salivary glands of diabetic patients [6, 9–14].

Various imaging modalities are used to assess the morphology and volume of salivary glands [10]. While Computed Tomography (CT) is utilized particularly for evaluating salivary gland stones, calcifications, and tumoral formations, it is not preferred in routine practice due to its use of ionizing radiation [15, 16]. Ultrasonography (USG) is frequently chosen as the first-line imaging modality for visualizing salivary glands because it is non-invasive, does not involve ionizing radiation, and is readily accessible [10]. However, USG may have limitations in evaluating deeply located structures and in volumetric assessment. Sialography, although a specific method allowing evaluation of salivary gland ducts, is an invasive procedure which limits its use. Magnetic Resonance (MR) imaging plays a significant role in the structural and volumetric assessment of salivary glands due to its high soft-tissue resolution, lack of ionizing radiation, and multiplanar imaging capability [16]. The use of MR imaging for the volumetric assessment of salivary glands is increasingly widespread, particularly because of its superior soft-tissue contrast, and it is considered the gold standard for evaluating the parotid gland [17].

In addition to volumetric measurements, histogram analysis has emerged as a quantitative imaging method for evaluating tissue characteristics based on the distribution of signal intensities within a region of interest. Parameters such as mean grayscale value, skewness, and kurtosis may help detect subtle microstructural alterations that are not apparent on conventional morphologic assessment [18, 19]. Previous studies have shown the potential of histogram analysis in identifying diffuse tissue changes associated with systemic conditions [20]. Therefore, combining histogram analysis with MR-based volumetric assessment may provide a more comprehensive evaluation of diabetes-related salivary gland involvement.

In this retrospective study, MR imaging was used to investigate both volumetric and histogram-based characteristics of the parotid and submandibular glands in patients with type 2 diabetes mellitus (T2DM). The findings were compared with those of control participants to improve understanding of T2DM-related salivary gland involvement and its potential implications for oral health.

## Materials and methods

Following approval from the Harran University Clinical Research Ethics Committee (Date: 10.03.2025, No: HRÜ/25.05.41), this retrospective study involved a review of previously acquired MR images from patients who had presented to the Harran University School of Medicine for various reasons. Participants aged 18 years or older with T1-weighted MR images of sufficient diagnostic quality allowing evaluation of the bilateral parotid and submandibular glands were eligible. For the T2DM group, eligibility required a previously established clinical diagnosis of T2DM documented in the electronic medical records. Established diagnostic criteria for diabetes include fasting plasma glucose ≥ 126 mg/dL, 2-h plasma glucose ≥ 200 mg/dL during a 75-g oral glucose tolerance test, HbA1c ≥ 6.5%, or random plasma glucose ≥ 200 mg/dL in the presence of classic symptoms of hyperglycemia [21]. Because of the retrospective design, these diagnostic thresholds were not re-applied by the investigators, and diabetes was not newly diagnosed or reclassified for study purposes. Patients with type 1 diabetes or other specific types of diabetes were not included. Control participants were selected from individuals with no documented systemic disease in the available medical records; HbA1c data were not available for control-group selection. Patients with imaging artifacts affecting gland assessment, salivary gland tumors, Sjögren’s syndrome or other known autoimmune salivary gland diseases, acute inflammatory conditions involving the salivary glands, previous salivary gland surgery, or a history of head and neck radiotherapy/chemotherapy were excluded.

An a priori power analysis was performed using G*Power 3.1, based on the between-group difference in parotid gland volume reported by Erdem and Tosun [14]. Based on the reported values, the effect size was calculated as Cohen’s d = 0.96. Using a two-tailed independent-samples t test, an alpha level of 0.05, a statistical power of 95%, and an allocation ratio of 1:1, the minimum required sample size was determined to be 60 participants (30 per group). Accordingly, the final study population consisted of 30 patients with T2DM and 30 control participants.

A 1.5 Tesla MR scanner (Magnetom Symphony; Siemens, Erlangen, Germany) was used for image acquisition. For axial T1-weighted images, the acquisition parameters were as follows: TR, 549 ms; TE, 8.7 ms; slice thickness, 4 mm; matrix, 315 × 320 pixels; field of view, 240 × 337 mm; and 32 slices. For axial T2-weighted images, the corresponding parameters were TR, 3600 ms; TE, 81 ms; slice thickness, 4 mm; matrix, 174 × 288 pixels; field of view, 240 × 206 mm; and 32 slices. Because this was a retrospective study, the analyses were restricted to the standardized non-contrast axial T1-weighted sequence consistently available across participants. Contrast-enhanced MRI was not evaluated because enhancement characteristics were outside the scope of the present study. The volumes of the parotid and submandibular salivary glands were evaluated on axial T1-weighted images using the Sectra IDS7 software program on a full HD screen with a maximum resolution of 1920 × 1080 and a screen size of 15.6 inches.

**Fig. 1:**
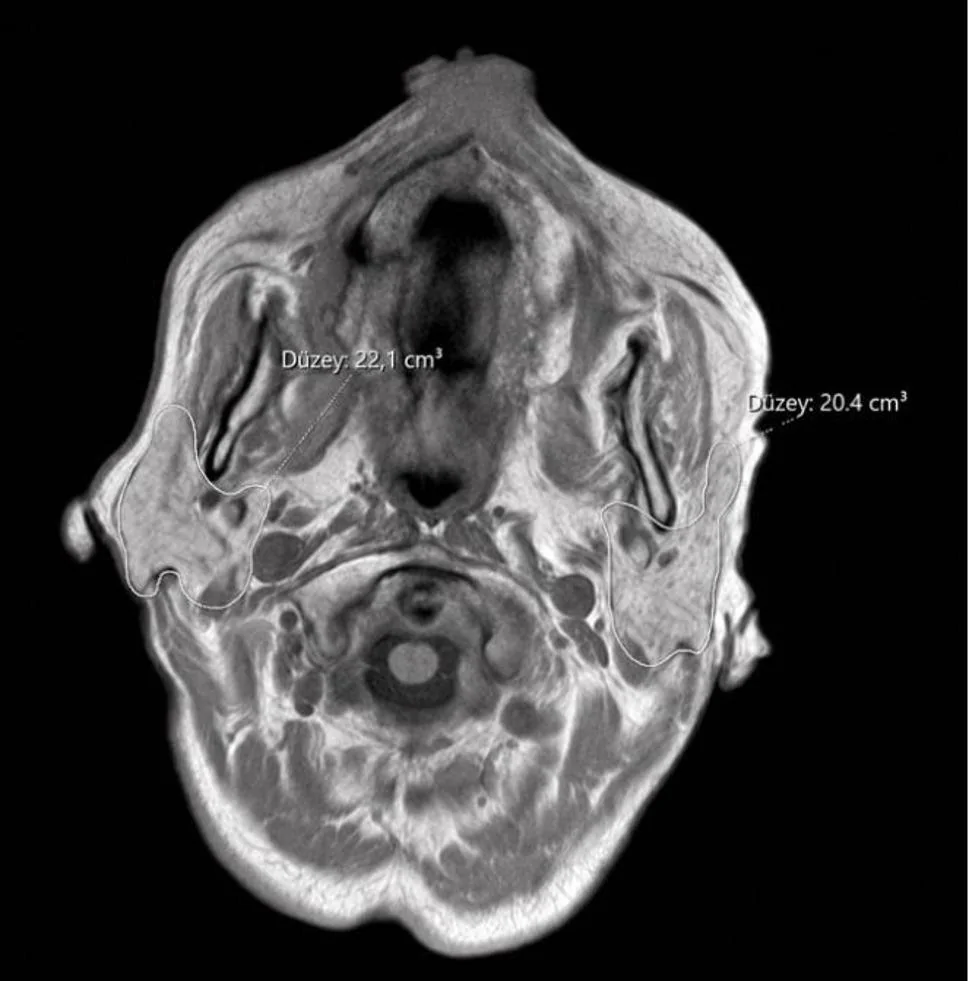
Manual delineation of the parotid gland boundaries and calculation of the parotid gland volume on axial T1-weighted MR imaging

**Fig. 2:**
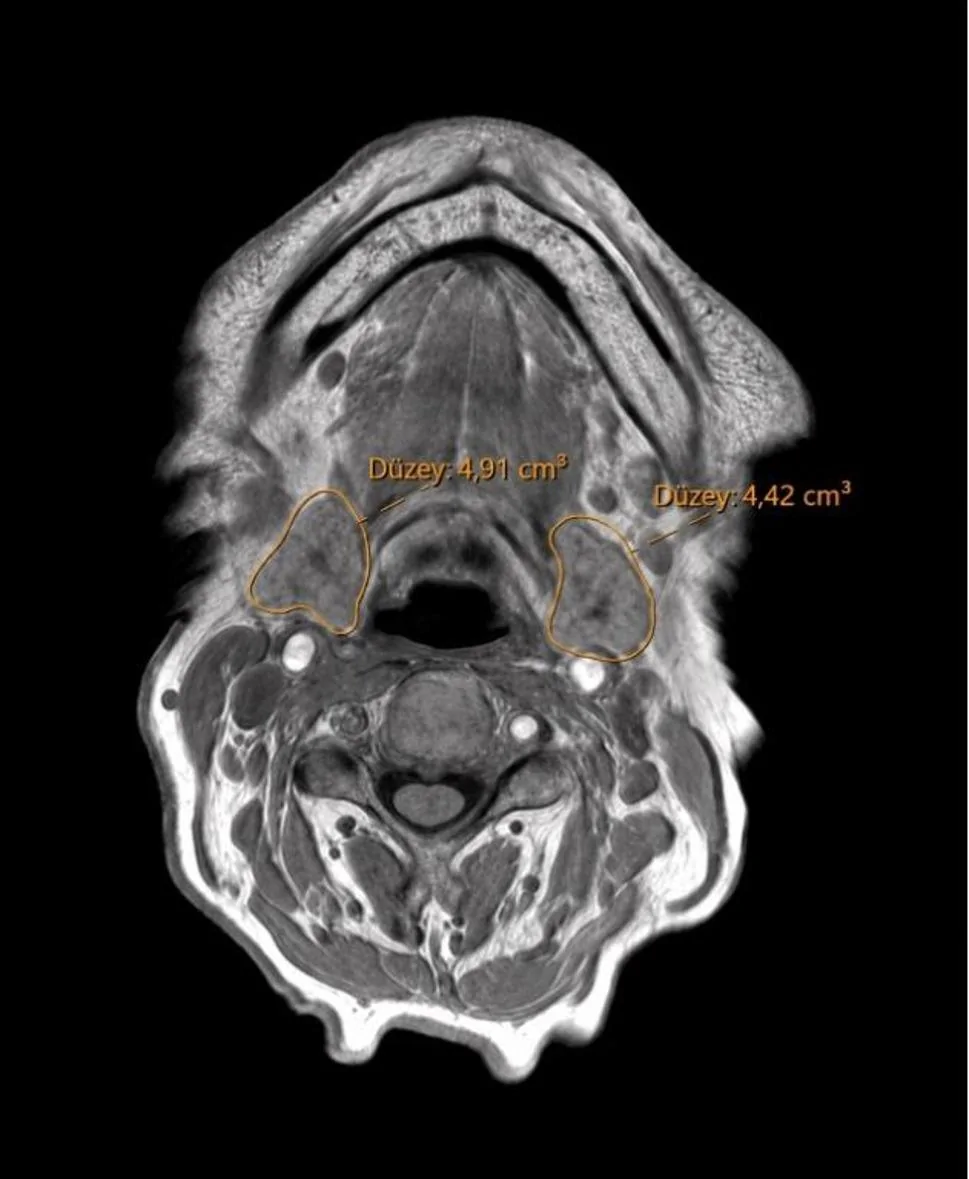
Manual delineation of the submandibular gland boundaries and calculation of the submandibular gland volume on axial T1-weighted MR imaging

**Fig. 3:**
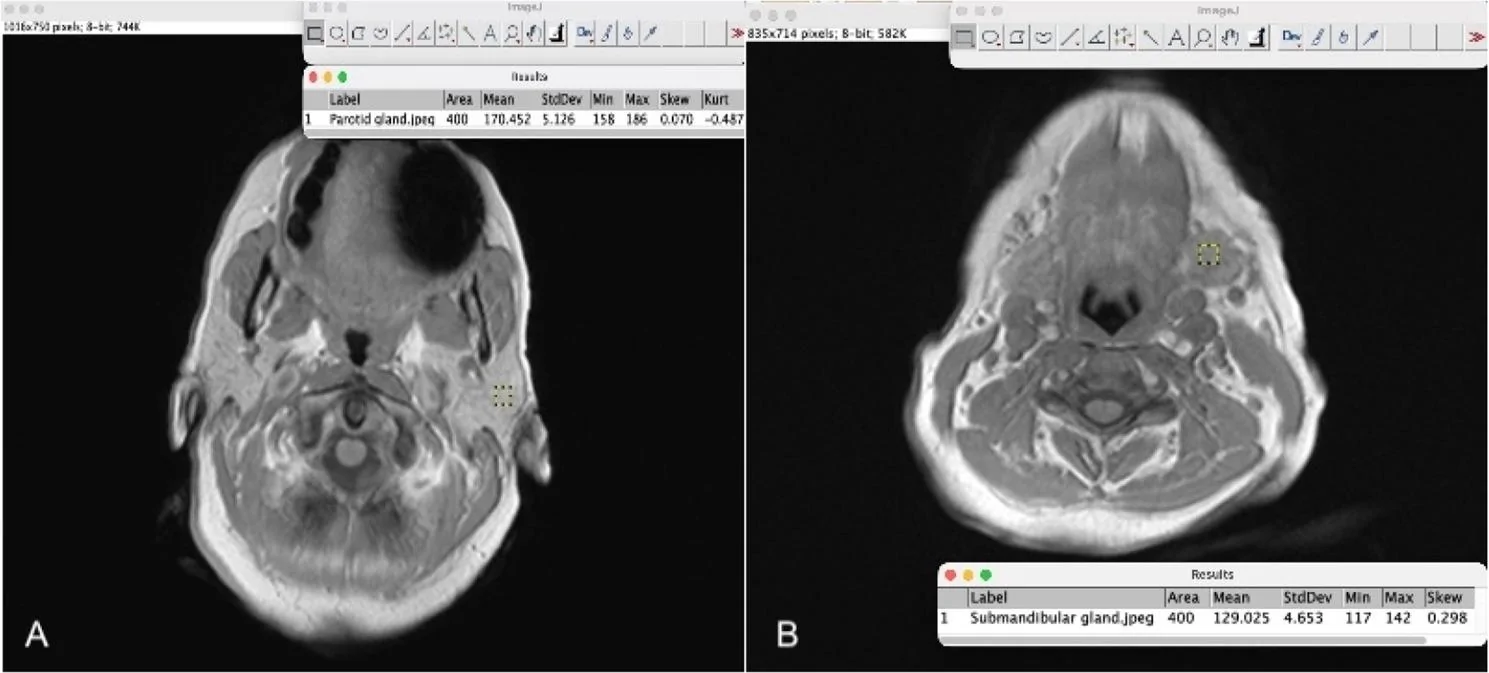
Representative axial T1-weighted MR images illustrating standardized ROI placement for histogram analysis using ImageJ. (**A**) A 20 × 20-pixel ROI positioned within the parotid gland parenchyma. (**B**) A 20 × 20-pixel ROI positioned within the submandibular gland parenchyma. Mean grayscale value, skewness, and kurtosis were calculated from the defined ROIs

For volumetric assessment, the parotid and submandibular glands were manually delineated on consecutive axial T1-weighted images throughout the entire visible extent of each gland. The right and left glands were segmented separately. Non-glandular structures, including lymph nodes, blood vessels, and extracapsular fat, were excluded from the delineation. The resulting segmentation was reviewed in the other imaging planes, and manual corrections were performed when necessary to refine the gland boundaries. Total gland volume was then calculated by the software from the complete set of delineated slices. Thus, volumetric assessment represented whole-gland, slice-by-slice segmentation rather than estimation from a single or selected section. (Figures 1 and 2). The age, gender, and volume measurements of all participants were recorded. For histogram analysis, T1-weighted MR images were exported to ImageJ software (National Institutes of Health, Bethesda, MD, USA) and converted to an 8-bit grayscale format. A single axial T1-weighted image was selected for each gland at the level where the glandular parenchyma was most clearly visualized and could be reliably distinguished from adjacent structures. A standardized 20 × 20-pixel square ROI was manually positioned within the glandular parenchyma. The fixed ROI size was used to standardize the sampled area across participants while avoiding visible ducts, vascular structures, surrounding adipose tissue, and other non-parenchymal components. In the parotid glands, the ROI was placed within the superficial lobe. Representative examples of ROI placement in the parotid and submandibular glands are shown in Fig. 3. Histogram-derived parameters, including mean grayscale value, skewness, and kurtosis, were automatically generated for each ROI.

All image analyses were conducted by an experienced dentomaxillofacial radiologist. For intra-observer reliability assessment, volumetric and histogram measurements were each repeated in 20 cases after a two-week interval. During the repeat evaluation, the observer was unaware of the initial measurements. Intra-observer reliability was assessed using a two-way mixed-effects intraclass correlation coefficient (ICC) with absolute agreement for single measurements, together with 95% confidence intervals (CIs). ICC values greater than 0.90 were considered indicative of excellent reliability.

### Statistical analysis

All statistical analyses were conducted using IBM SPSS Statistics version 24.0 (IBM Corp., Armonk, NY, USA). The normality of continuous variables was evaluated using both visual methods (histograms and Q-Q plots) and analytical methods (Shapiro-Wilk test). Descriptive statistics are reported as mean ± standard deviation for continuous variables and as frequencies (n) for categorical variables. An independent samples t-test was used to compare the age between the T2DM and control groups, while the chi-square test was employed to assess differences in gender distribution. The comparison of right and left parotid and submandibular salivary gland volumes between the two groups was performed using the Independent Samples t-test. Since histogram parameters were not normally distributed, comparisons of mean grayscale values, skewness, and kurtosis between groups were performed using the Mann–Whitney U test. Within the T2DM group, the relationship between HbA1c levels and salivary gland volumes (right parotid, left parotid, right submandibular, left submandibular) was assessed using Pearson correlation analysis. Furthermore, the relationships between age and salivary gland volumes in both groups were examined separately using Pearson correlation analysis. The relationship between disease duration and parotid/submandibular salivary gland volumes in the T2DM group was also analyzed using the same method. The statistical significance level was set at *p* < 0.05. For the 12 comparisons involving the three histogram parameters across the four salivary glands, a Bonferroni correction was applied to account for multiple testing, resulting in an adjusted significance threshold of *p* < 0.0042. All p-values were two-tailed. For the interpretation of correlation coefficients, the Pearson correlation coefficient (r) values were classified as follows: ±0.70–1.00 (strong), ± 0.30–0.69 (moderate), and ± 0.00–0.29 (weak).

## Results

All 30 patients in the diabetes group had a previously established diagnosis of T2DM. The mean age of the individuals in the T2DM group was 61.43 ± 15.01 years, while it was 56.97 ± 13.22 years in the control group. There was no statistically significant difference in the mean age between the two groups (*p* = 0.226). Regarding gender distribution, the T2DM group consisted of 15 male and 15 female individuals, while the control group consisted of 14 male and 16 female individuals. No significant difference was found in gender distribution between the groups (*p* = 0.796). The mean HbA1c value in the T2DM group was 8.14 ± 1.78%; HbA1c data was not available for the control group in the retrospective records used for this study. The mean disease duration in the T2DM group was 12.70 ± 6.54 years (Table 1).

**Table 1:** Comparison of demographic and clinical characteristics between T2DM and control groups

Characteristic	T2DM Group (*n* = 30)	Control Group (*n* = 30)	*p*-value
Age (years)	61.43 ± 15.01	56.97 ± 13.22	0.226
Gender (M/F)	15/15	14/16	0.796
HbA1c (%)	8.14 ± 1.78	NA	NA
DM Duration (years)	12.70 ± 6.54	NA	NA

Age data are presented as mean ± standard deviation. Gender data are presented as numbers (n). Intergroup age comparison was performed using the Independent Samples t-test, and gender distribution comparison was performed using the Chi-square test. *T2DM* Type 2 Diabetes Mellitus, *NA* Not Available

When comparing the parotid and submandibular salivary gland volumes of the T2DM and control groups, no statistically significant difference was observed. The right parotid gland volumes of individuals in the T2DM group (Mean ± SD: 21.18 ± 4.43 cm³) were similar to those of the control group (20.68 ± 6.22 cm³), and the difference was not significant (*p* = 0.724). Similarly, the left parotid gland volumes were 20.93 ± 4.34 cm³ in the T2DM group and 20.34 ± 5.63 cm³ in the control group, with no significant difference between the groups (*p* = 0.654). Analysis of the submandibular gland volumes revealed values of 7.26 ± 1.79 cm³ for the right submandibular gland in the T2DM group versus 7.38 ± 1.43 cm³ in the control group (*p* = 0.770), and 7.15 ± 2.03 cm³ for the left submandibular gland in the T2DM group versus 7.41 ± 1.45 cm³ in the control group (*p* = 0.566). No statistically significant difference was detected between the groups for either gland (Table 2).

**Table 2:** Comparison of parotid and submandibular salivary gland volumes in T2DM and control groups

Variable (cm³)	T2DM Group (*n* = 30)	Control Group (*n* = 30)	*p*-value
Right Parotid	21.18 ± 4.43	20.68 ± 6.22	0.724
Left Parotid	20.93 ± 4.34	20.34 ± 5.63	0.654
Right Submandibular	7.26 ± 1.79	7.38 ± 1.43	0.770
Left Submandibular	7.15 ± 2.03	7.41 ± 1.45	0.566

Data are presented as mean ± standard deviation (SD). Intergroup comparisons were made using the Independent Samples t-test. A p-value < 0.05 was considered statistically significant. Volume unit is cubic centimeters (cm³). T2DM; Type 2 Diabetes Mellitus

Analysis of the relationship between HbA1c levels and parotid/submandibular salivary gland volumes in the T2DM group revealed that all correlations were negative in direction; however, none reached statistical significance. A negative correlation was found between right parotid gland volume and HbA1c (*r* = -0.334; *p* = 0.071). Similarly, a weak negative correlation was detected between left parotid gland volume and HbA1c (*r* = -0.265; *p* = 0.156). For the submandibular glands, a weak negative correlation was observed on the right side (*r* = -0.150; *p* = 0.429) and a similarly weak negative correlation on the left side (*r* = -0.236; *p* = 0.210) (Table 3).

**Table 3:** Correlation analysis between HbA1c and salivary gland volume parameters

Variable (cm³)	*r*	*p*-value
Right Parotid	-0.334	0.071
Left Parotid	-0.265	0.156
Right Submandibular	-0.150	0.429
Left Submandibular	-0.236	0.210

Pearson correlation analysis was used. All correlations were negative in direction and were not statistically significant (*p* > 0.05). *p*-values are based on two-tailed tests. r: correlation coefficient

Analysis of the relationship between DM duration and salivary gland volumes in the DM group revealed that all correlations were negative in direction; however, none reached statistical significance. A weak negative correlation was found between right parotid gland volume and DM duration (*r* = -0.095; *p* = 0.616). The relationship between left parotid gland volume and DM duration was similarly weak and non-significant (*r* = -0.041; *p* = 0.829). For the submandibular glands, negative but statistically non-significant correlations were observed on both the right (*r* = -0.219; *p* = 0.244) and left (*r* = -0.219; *p* = 0.245) sides (Table 4).

**Table 4:** Correlation analysis between diabetes mellitus duration and salivary gland volume parameters

Variables (*n* = 30)	*r*	*p*-value
Right Parotid (cm³)	-0.095	0.616
Left Parotid (cm³)	-0.041	0.829
Right Submandibular (cm³)	-0.219	0.244
Left Submandibular (cm³)	-0.219	0.245

Pearson correlation analysis was used. No statistically significant relationship was detected between any variables (*p* > 0.05). *p*-values are based on two-tailed tests. r: correlation coefficient

Table 5 presents the histogram parameters of the major salivary glands in the T2DM and control groups. Mean grayscale values were significantly lower in all major salivary glands of patients with DM than in controls, and all differences remained statistically significant after Bonferroni correction (adjusted significance threshold, *p* < 0.0042). No significant differences were observed in skewness or kurtosis values between the groups. Intra-observer reliability was excellent for all volumetric and histogram measurements, with ICC values ranging from 0.9763 to 0.9999. Individual ICC values and corresponding 95% confidence intervals are presented in Table 6.

**Table 5:** Comparison of salivary gland histogram parameters between T2DM and control groups

Parameter	Group	*n*	Mean Rank	Test Statistic	*P*-value
MEAN					
Right parotid	T2DM	30	23.03	U = 226.0	0.002*
	Control	30	37.21		
Left parotid	T2DM	30	23.50	U = 240.0	0.003*
	Control	30	36.72		
Right submandibular	T2DM	30	23.03	U = 226.0	0.002*
	Control	30	37.21		
Left submandibular	T2DM	30	22.13	U = 199.0	< 0.001*
	Control	30	38.14		
SKEWNESS					
Right parotid	T2DM	30	32.12	U = 371.5	0.336
	Control	30	27.81		
Left parotid	T2DM	30	30.58	U = 417.5	0.791
	Control	30	29.40		
Right submandibular	T2DM	30	30.72	U = 413.5	0.744
	Control	30	29.26		
Left submandibular	T2DM	30	27.03	U = 346.0	0.177
	Control	30	33.07		
KURTOSIS					
Right parotid	T2DM	30	29.77	U = 428.0	0.915
	Control	30	30.24		
Left parotid	T2DM	30	30.23	U = 428.0	0.915
	Control	30	29.76		
Right submandibular	T2DM	30	29.20	U = 411.0	0.716
	Control	30	30.83		
Left submandibular	T2DM	30	32.90	U = 348.0	0.187
	Control	30	27.00		

Data are presented as mean ranks. Intergroup comparisons were performed using the Mann–Whitney U test. A Bonferroni-corrected significance threshold of *p*  < 0.0042 was applied to the 12 histogram comparisons. *T2DM* Type 2 diabetes mellitus

*Statistically significant after Bonferroni correction (*p* < 0.0042)

**Table 6:** Intra-observer reliability of volumetric and histogram measurements

Salivary gland	Volume ICC (95% CI)	Mean grayscale ICC (95% CI)	Skewness ICC (95% CI)	Kurtosis ICC (95% CI)
Right parotid	0.9984 (0.9939–0.9996)	0.9991 (0.9966–0.9998)	0.9998 (0.9991–0.9999)	0.9999 (0.9994–1.0000)
Left parotid	0.9992 (0.9970–0.9998)	0.9991 (0.9963–0.9998)	0.9995 (0.9982–0.9999)	0.9997 (0.9986–0.9999)
Right submandibular	0.9763 (0.9084–0.9941)	0.9992 (0.9969–0.9998)	0.9995 (0.9980–0.9999)	0.9997 (0.9990–0.9999)
Left submandibular	0.9951 (0.9325–0.9990)	0.9997 (0.9989–0.9999)	0.9994 (0.9979–0.9999)	0.9997 (0.9989–0.9999)

*ICC, *intraclass correlation coefficient; *CI, *confidence interval. ICC values are presented with corresponding 95% CIs. Intra-observer reliability was assessed in 20 cases.

## Discussion

The effects of DM on salivary glands are discussed in the literature with varying results concerning morphological and functional parameters such as gland volume, echogenicity, elasticity, and vascularity [10, 12–14]. Particularly, MRI-based volumetric studies are limited in number.

Existing literature reports that in diabetic individuals, parotid and submandibular gland volumes may increase or at least be preserved, potentially due to sialadenosis, fibrosis, fat infiltration, or microvascular changes [10, 12, 13, 17, 22]. However, the data obtained in our study did not fully align with this general trend. No statistically significant difference was found between the T2DM and control groups regarding both parotid and submandibular gland volumes. Furthermore, no significant correlation was observed between HbA1c levels and salivary gland volumes in the diabetic group. The lower mean volume values observed in the submandibular glands also did not reach statistical significance.

Our findings differed from previous USG-based studies reporting increased salivary gland size or volume in patients with T2DM [10, 12, 13]. Öztürk and Yalçın [12] observed higher parotid and submandibular gland dimensions and volumes in patients with DM, with greater values in type 2 than in type 1 DM, whereas Gupta et al. [10] reported a positive association between HbA1c levels and ultrasonographic parotid gland dimensions. In contrast, no significant association between HbA1c and gland volume was identified in the present study. Although all correlation coefficients were negative in direction, none reached statistical significance; therefore, this pattern should not be interpreted as evidence of an inverse relationship between HbA1c and salivary gland volume. These discrepancies may partly reflect differences in imaging modality and measurement methodology, as previous studies predominantly used USG-based dimensional or volumetric measurements, whereas the present study employed whole-gland, slice-by-slice MRI-based volumetry. The use of T1-weighted MRI for salivary gland volumetry is also supported by previous population-based research; Brzoska et al. [17] used non-contrast T1-weighted MRI to assess parotid gland volume in a large cohort of 1,725 individuals. In the present study, the standardized axial T1-weighted sequence available for all participants provided a uniform imaging basis for both volumetric and histogram analyses. Differences in diabetes subtype across studies, glycemic control, disease duration, treatment, and accompanying metabolic factors may also contribute to variability among studies. Recent USG findings similarly indicate increased salivary gland dimensions in patients with T2DM, while emphasizing the need to consider HbA1c, disease duration, and treatment-related factors when interpreting structural glandular changes [23].

In the analyses examining the relationship between DM duration and salivary gland volumes, all correlations were negative in direction, but these relationships did not reach statistical significance. This suggests that structural deteriorations (e.g., atrophy, fibrosis) that may develop in the glandular tissue with disease progression might not be detectable volumetrically in the early stages. Indeed, the study by Erdem and Tosun [14] on microvascular structures demonstrated a negative correlation between DM duration and decreased microvascularity.

Although manual MR volumetry used in our study provides higher tissue resolution and offers improved soft-tissue delineation and enables whole-gland volumetric assessment, variations due to the measurement technique/software constitute a potential limitation. Furthermore, heterogeneity in glycemic control, medication use, metabolic comorbidities, and other unmeasured clinical factors may also have contributed to variability in glandular measurements and MR signal characteristics.

Lindeberg and Andersen [22] reported that the increased gland volume detected in diabetic individuals resulted from an increase in fibrotic and adipose tissue despite parenchymal cell loss. This indicates that volumetric measurements alone may not fully reflect the underlying parenchymal composition or tissue characteristics of the gland.

In addition to volumetric assessment, histogram analysis demonstrated significantly lower mean grayscale values in all major salivary glands of patients with T2DM, whereas skewness and kurtosis did not differ significantly between groups. This pattern indicates a group-level difference in T1-weighted signal intensity while the overall shape of the signal-intensity distribution remained relatively preserved. A comparable pattern was reported by Kotanlı et al. [20], who observed lower mean grayscale values in the parotid glands of hypertensive patients without significant changes in skewness or kurtosis. Taken together, these findings may be compatible with a relatively diffuse shift in the sampled T1-weighted signal-intensity distribution rather than increased focal heterogeneity. However, unchanged skewness and kurtosis should not be interpreted as direct evidence of diffuse microstructural pathology, particularly given the nonspecific nature of conventional T1-weighted signal intensity.

Conventional T1-weighted grayscale values are not tissue-specific quantitative parameters; therefore, the histopathological basis of the observed signal differences cannot be determined from the present findings. On T1-weighted imaging, tissues with shorter T1 relaxation times generally exhibit higher signal intensity, and adipose tissue typically appears hyperintense. Accordingly, increased fatty infiltration alone would not readily explain the lower grayscale values observed in the T2DM group. Changes in tissue water, macromolecular composition, or other aspects of tissue architecture may also influence T1-weighted signal, but these effects cannot be attributed specifically to fibrosis, acinar alterations, or other histopathological processes. In addition, conventional MR signal intensity is affected by proton density, acquisition parameters, receiver-coil characteristics, and image scaling. Notably, Merlo et al. found no significant difference between diabetic and control parotid glands in the proportion of stromal fatty tissue [24].

Previous histopathological and clinical studies have described several alterations in diabetic salivary glands, including acinar changes, fibrosis, adipose tissue infiltration, microvascular impairment, and extracellular matrix remodeling [1, 4, 22, 25]. Although these mechanisms may provide biological context for the present imaging observations, they should not be interpreted as directly demonstrated by our findings. Therefore, the lower grayscale values observed in the DM group are more appropriately regarded as an exploratory imaging finding whose biological significance requires confirmation using quantitative MRI techniques and histopathological or functional correlation.

Previous studies have shown that structural alterations in diabetic salivary glands may coexist with functional and biochemical abnormalities. Carda et al. [25] reported increased salivary urea and total protein levels, decreased microalbumin levels, and increased salivary glucose in patients with poor glycemic control. Dry mouth was observed in 76.4% of patients, while tooth and/or gingival disease was reported in 100%. Histopathological examination also revealed structural alterations in the parotid gland, including smaller acini and adipose infiltration [25]. However, as salivary function and biochemical parameters were not evaluated in the present study, these findings cannot be directly correlated with our imaging results.

Our study has several limitations. These include its retrospective design, limited sample size, and the lack of detailed or uniformly available data on potential confounding factors, including body mass index (BMI), hypertension, dyslipidemia, metabolic syndrome, diabetes-related complications, detailed antidiabetic and potentially xerogenic medication use, alcohol consumption, hydration status, and smoking. Therefore, residual confounding cannot be excluded. Moreover, the a priori sample size calculation was performed based on the expected between-group difference in parotid gland volume rather than histogram-derived parameters. Thus, the histogram results are to be considered exploratory and await confirmation in prospective studies with adequate power for these endpoints. In addition, HbA1c measurements were unavailable for the control group, and unrecognized prediabetes or diabetes cannot be completely excluded. Salivary gland function was not directly assessed using salivary flow measurements, xerostomia questionnaires, or biochemical analyses; therefore, the functional significance of the imaging findings could not be determined. Furthermore, the study was not designed to evaluate the diagnostic performance or clinical utility of the histogram parameters.

The manual nature of the measurements may also introduce observer-dependent variability, while the single-slice fixed-size ROI approach may be susceptible to sampling bias and may not fully capture whole-gland spatial heterogeneity. Although all images were acquired on the same MRI system using a standardized protocol, no internal-reference or phantom-based gray-level normalization was performed. Therefore, conventional T1-weighted signal-intensity differences should be interpreted as relative rather than absolute quantitative tissue measurements, and interindividual differences in signal-intensity scaling may have influenced the grayscale measurements. Histopathological validation was unavailable to determine the tissue-level basis of these differences. Although excellent intra-observer reliability was demonstrated, inter-observer reliability could not be assessed because all image analyses were performed by a single observer. Smoking has been reported to cause structural and functional changes in the salivary glands, including increased gland volume, parenchymal heterogeneity, altered vascularity and increased tissue stiffness [26]. Smoking was not evaluated as a study variable; therefore, its potential confounding effect on salivary gland morphology and MR signal characteristics cannot be excluded. Future studies should incorporate detailed smoking histories to more clearly determine the independent impact of diabetes on salivary gland morphology and microstructure.

Future prospective studies with larger sample sizes should prioritize the standardized recording of factors such as BMI, glycemic control, and medication therapy. Whole-gland three-dimensional segmentation with volumetric histogram or radiomic analysis would also be valuable for determining whether the observed signal-intensity differences are reproducible throughout the entire gland. Moreover, future studies incorporating quantitative and multiparametric MRI techniques, including DWI/ADC mapping, Dixon-based fat fraction imaging, MR spectroscopy, and dynamic contrast-enhanced MRI, may provide complementary information regarding tissue diffusion, fat content, metabolic composition, and perfusion, respectively, and may contribute to a more comprehensive characterization of diabetic salivary gland alterations.

## Conclusion

The results of this study showed no statistically significant difference in parotid and submandibular salivary gland volumes between patients with T2DM and control participants. In addition, within the T2DM group, no significant correlation was found between HbA1c levels or diabetes duration and gland volumes. However, histogram analysis revealed lower mean grayscale values across all major salivary glands in the T2DM group, whereas skewness and kurtosis parameters remained unchanged. These findings represent group-level differences in T1-weighted signal intensity that were not accompanied by measurable volumetric differences. However, their biological and clinical significance remains uncertain, and they should not be interpreted as evidence of salivary gland dysfunction or as clinically validated imaging biomarkers. Further studies incorporating quantitative MRI techniques and functional assessment are needed to clarify the significance of these findings.

## Data Availability

The datasets generated and/or analysed during the current study are not publicly available due to privacy considerations associated with retrospective clinical data but are available from the corresponding author on reasonable request.
